# Unusual Fungal Endocarditis Causing Disseminated Infection After Renal Transplant

**DOI:** 10.7759/cureus.38896

**Published:** 2023-05-11

**Authors:** Waleed Ali, Bradley Casey, Ilya Al Salman, Haitham Mazek, Rahel Alemu, Usman Younus

**Affiliations:** 1 Cardiology, Cape Fear Valley Medical Center, Fayetteville, USA; 2 Internal Medicine, Cape Fear Valley Medical Center, Fayetteville, USA

**Keywords:** kidney transplant recipient, left ventricular outflow obstruction (lvot), lvot, of pseudoallescheria boydii, antimicrobial resistance infectious diseases

## Abstract

Fungal endocarditis is a relatively uncommon disease; it mostly affects those with intracardiac devices and those with compromised immune systems. Scedosporium apiospermum (S. apiospermum), the asexual state of Pseudoallescheria boydii, has become increasingly reported as an opportunistic pathogen. These filamentous fungi present in soil, sewage, and polluted waters, and was previously recognized to cause human infection after their inhalation or traumatic subcutaneous implantation. In immunocompetent individuals, it usually causes localized diseases depending on the site of entry such as skin mycetoma. However, in immunocompromised hosts, the fungus species appear to disseminate and cause invasive infections, frequently reported to be life-threatening with poor response to antifungal medications. S. apiospermum invasive endocarditis remains a rare complication, mostly cited in immunocompetent hosts with prosthetic cardiac valves or other intracardiac devices and severely immunocompromised patients with hematologic neoplasia. Herein, we describe the case of a renal transplant patient on immunosuppressive medications who presented with S. apiospermum fungal septic infection that invaded the left ventricular outflow tract (LVOT) causing endocarditis with disseminated infection and resulted in poor clinical outcome.

## Introduction

Fungal endocarditis is infrequently encountered in clinical practice and thus requires a high index of suspicion to avoid poor clinical outcomes. The disease usually presents as subacute endocarditis and has an insidious course, especially among those who are immunocompromised. Prolonged fever associated with sweating and fatigue is the most common presentation. However, in immunocompromised hosts, the disease process may go unrecognized and present with clinical signs of septic embolization to the brain, kidneys, peripheral circulation, and gastrointestinal tract. We report a case of a renal transplant patient on immunosuppressive medications who presented with symptoms concerning for acute cerebrovascular accident; he was later found to have brain-septic emboli with a large fungal mass that invaded the left ventricular outflow tract (LVOT). The clinical course was challenging and detoured unexpectedly to a poor clinical outcome.

## Case presentation

**Figure 1:**
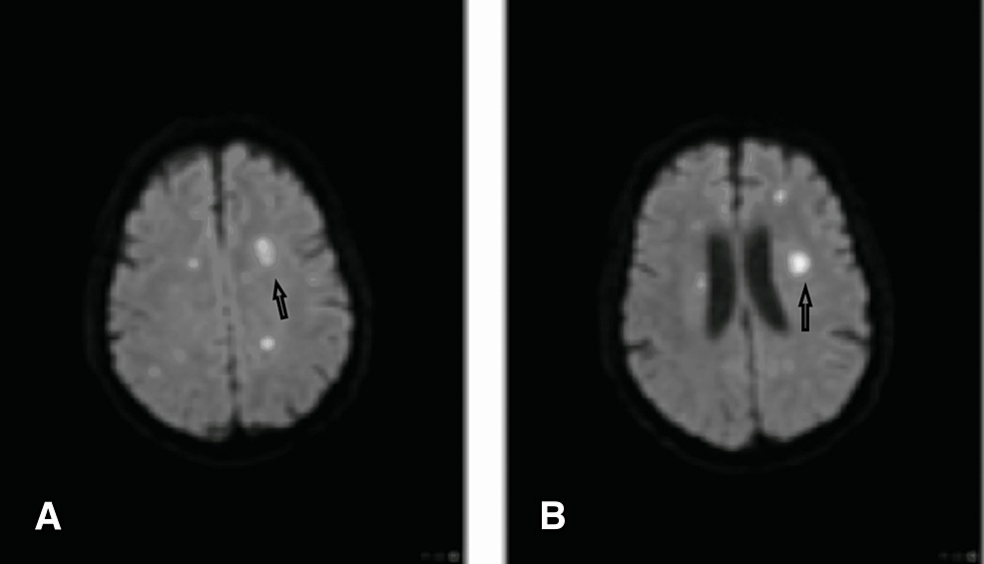
MRI diffusion-weighted imaging of the brain showing multiple foci of restricted diffusion; most lesions involved the basal ganglia and brain stem (arrows).

A 67-year-old male with a past medical history of end-stage renal disease (ESRD) secondary to hypertension and obstructive nephropathy had recently undergone a deceased donor kidney transplant six months prior to admission. He received induction treatment with solumedrol and thyroglobulin followed by triple immunosuppression therapy with prednisone, mycophenolate, and tacrolimus. Post-transplant, he developed delayed graft rejection and was restarted on hemodialysis. Despite delayed graft rejection, the patient was continued on triple immunosuppression since he was being relisted for a second kidney transplant. The patient presented to our facility with a chief complaint of ataxia and double vision. Two days prior to his presentation, he underwent septoplasty at a local ear, nose, and throat (ENT) facility which was uneventful. The patient had two dissolvable nasal packings at the time of his presentation. Nasal irrigation with suctioning to washout packing was performed with no improvement in his symptoms. Physical examination was unremarkable. Initial work-up showed normal white cell count and stable chemistry except for elevated blood urea nitrogen and creatinine which was expected given his underlying ESRD. The patient underwent a head CT scan which was unremarkable. His clinical course detoured unexpectedly. He developed severe encephalopathy requiring endotracheal intubation for airway protection. He was started on vasopressor support to maintain adequate blood pressure. A brain MRI was performed which revealed numerous areas of restricted diffusion concerning for septic emboli with small internal hemorrhagic foci (Figure 1). 

**Figure 2:**
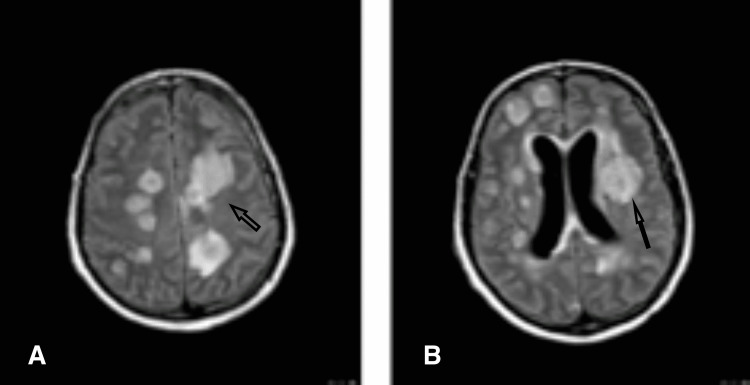
Brain fluid-attenuated inversion recovery (FLAIR) sequence showing multiple lesions with significant surrounding edema (arrows).

Given his clinical presentation, a transesophageal echocardiography (TEE) was obtained after a non-diagnostic transthoracic echocardiogram. TEE revealed a mass measuring 2.13 x 1.66 cm attached to the LVOT septal wall which appeared to be independent of the aortic valves (Figure 2).

The initial impression was possible cardiac tumor vs atypical vegetation vs a thrombus. The patient was started on broad-spectrum antibiotics including vancomycin and meropenem pending initial blood work up. His immunosuppressive medications were discontinued. His blood cultures remained negative despite running low-grade fevers. Further work up was positive for high fungitell level > 500 pg/ml (N < 80pg/ml), C-reactive protein (CRP) > 190 mg/dl (N < 3 mg/dl), and erythrocyte sedimentation rate (ESR) of 47 mm/hr (0-20 mm/hr.).

**Figure 3:**
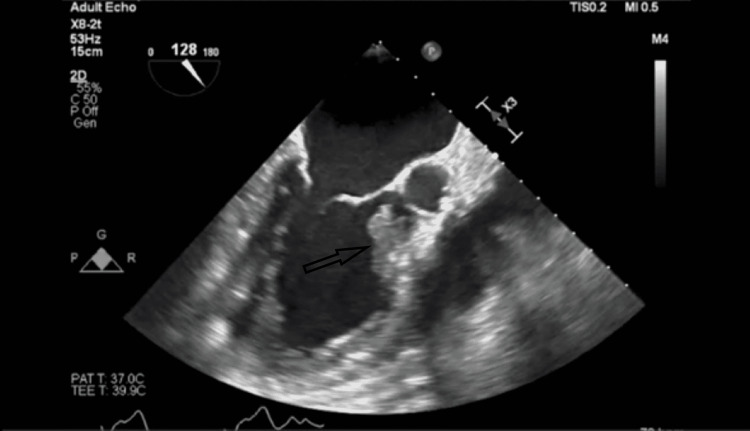
Transesophageal echocardiography; long axis view at 128 degrees showing the fungal ball protruding into the left ventricular outflow tract (LVOT) (arrow).

Cryptococcus screening and JC virus DNA PCR were negative. The patient was started on amphotericin 4mg/kg/per day due to the suspicion of fungal infection. A repeat brain MRI was performed later and it showed marked progression of his previous lesions which now involved the brainstem with worsening cerebral edema and effacement of sulci (Figure 3).

**Figure 4:**
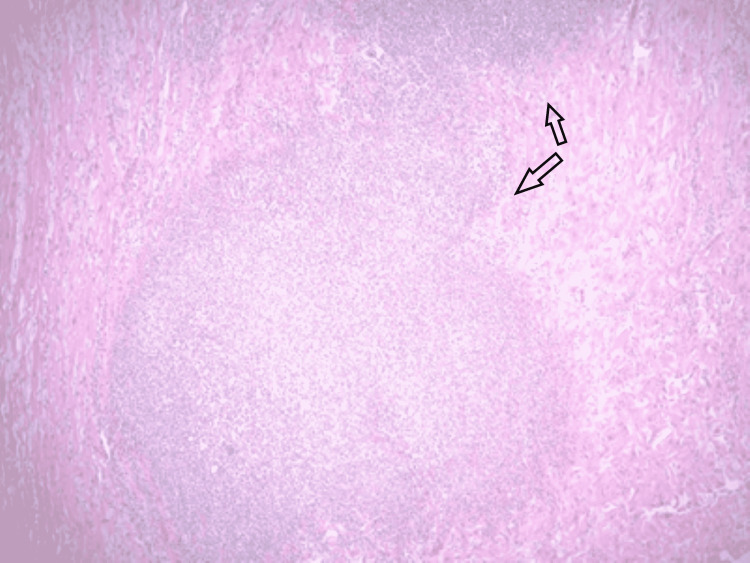
Light micrograph section through the heart tissue (LVOT) showing a small abscess within the myocardium (arrows). LVOT: left ventricular outflow tract

On day 5, he was noted to have no brain stem reflexes and no respiratory drive on the ventilator. He was declared brain dead after he failed the apnea test. Medical autopsy showed widespread fungal abscesses involving the myocardium, bilateral lungs, brain, and thyroid (Figure 4).

**Figure 5:**
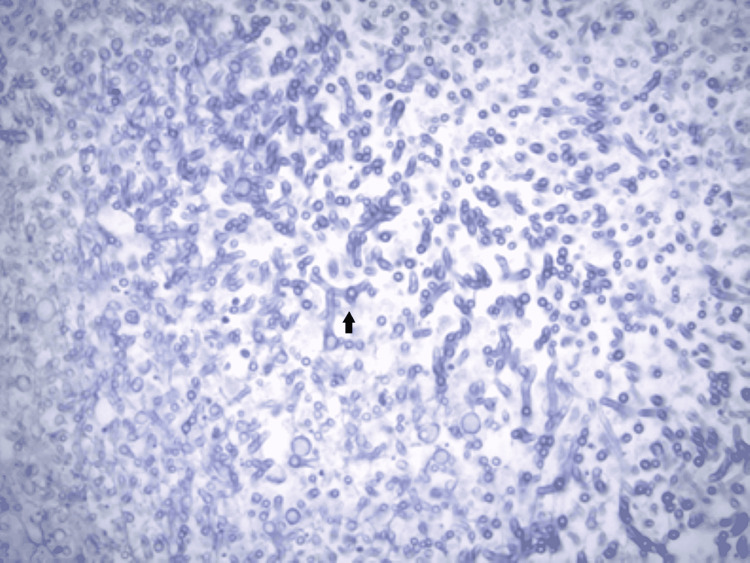
Light microscopy at 400x magnification showing fungal microorganisms (black arrow).

Swab cultures grew Scedosporium apiospermum (S. apiospermum) in the heart and bilateral lungs which confirmed complicated fungal endocarditis that resulted in a devastating clinical outcome (Figure 5). 

## Discussion

Fungal endocarditis is a rare but increasingly prevalent entity. It is usually seen in patients that are immunocompromised and/or receiving invasive interventions such as central venous catheters or prosthetic heart valves [1]. Candida and Aspergillus species are the most frequent pathogens reported in the literature, however, unusual organisms are frequently recognized. The two members of the Scedosporium genus, namely S. prolificans (formerly named S. inflatum) and S. apiospermum (the anamorph of Pseudoallescheria boydii) have been recognized as opportunistic pathogens with increased frequency in the current literature [2,3]. S. apiospermum, the asexual state of Pseudoallescheria boydii, are filamentous fungi present in soil, sewage, and polluted waters. It has been recognized to have five distinct species: S. apiospermum, S. boydii, S. dehoogii, S. aurantiacum, and S. minutisporum. Out of these five species, S. apiospermum, S. aurantiacum, and S. boydii are reported to cause human infections [2]. The clinical spectrum of diseases associated with these organisms was originally reported in immunocompetent individuals after their inhalation or traumatic subcutaneous implantation causing localized diseases depending on the site of entry such as skin ulcers, sinusitis, endophthalmitis, and osteomyelitis [3,4]. However, the clinical spectrum has expanded to include serious symptoms and disseminated diseases. Since the first reported serious disease in 1991, several cases have been reported exclusively in patients with leukemia and organ transplant recipients receiving immunosuppressive medications [5,6]. Native and prosthetic cardiac valve involvements carry worse outcomes and provide a nidus for septic emboli and disseminated infection [7]. Contrary to previously reported endocarditis, our case pertained to a unique presentation where the fungus invaded the LVOT with no evidence of native valve involvement. To our knowledge, this is the first reported case of endocarditis caused by S. apiospermum that invaded the LVOT in a renal transplant patient.

Although clinical diseases caused by these organisms bear no difference compared to other filamentous fungal infections, some characteristic features, albeit not pathognomonic, worth mentioning are a) cutaneous involvement with numerous skin nodules, often with a necrotic center; b) the tendency to involve the central nervous system causing brain abscess and meningoencephalitis; c) high incidence of endophthalmitis; d) extremely high incidence of tissue and blood cultures [8]. Likewise, other fungal infections, histopathological evidence, or positive culture are needed for a definitive diagnosis. In the present case, positive tissue cultures revealed rare fungal isolates.

Like in our case, these organisms tend to involve the central nervous system causing brain abscesses and meningoencephalitis [8]. Although fungal abscesses have a similar appearance to pyogenic brain abscesses in brain MRI, several studies investigated different brain MR clinical findings to establish diagnostic criteria that would improve clinical outcomes if appropriate treatment is administered early on, especially in those with a compromised immune system. Mueller-Mang and his colleagues reviewed the MR images from nine patients with fungal brain infections, and 17 patients with pyogenic brain abscess in a retrospective study to evaluate the role of DW images in distinguishing between fungal and bacterial brain abscesses [9]. The authors found that fungal brain abscesses tend to have higher apparent diffusion coefficient (ADC) values. Furthermore, other characteristics such as multiplicity, signal heterogeneity on T2-weighted and DW imaging, and involvement of deep grey-matter nuclei further support fungal brain infections. It is also worth noting that bacterial septic emboli typically demonstrate a rim enhancement while fungal infections tend to be centrally located in the brain as demonstrated in our case report.

Routine antifungal treatments in these cases hold poor outcomes due to the inherent resistance of this fungus to usual antifungal medications. This resulted in an almost 100% mortality rate among previous case reports, emphasizing the need for more effective therapeutic interventions. Nevertheless, Howden et al. reported successful control of disseminated Scedosporium fungal infection following a bone marrow transplant with a combination of voriconazole and terbinafine [10]. Likewise, Henao-Martínez et al. reported two cases of S. apiospermum central nervous system infections treated successfully with the combination of voriconazole and terbinafine [11]. This combination appears to be synergistic against this fungus in vitro, with evident synergism being found in 95% of the isolates in one study [11]. The mechanism of this synergy is not well understood, nevertheless, previous reports speculated several processes such as additive effects on different steps in the ergosterol biosynthesis pathway [11]. Moreover, emerging data suggest that micafungin with voriconazole demonstrate a potential therapeutic combination with a synergistic effect against several fungi including Scedosporium spp [11].

We believe that our patient encountered this rare fungal infection after his sinus surgery which likely resulted in subcutaneous implantation. His overall compromised immune system favored a disseminated infection. Unfortunately, our patient had a very rapid decline in clinical status before starting antifungal treatment, highlighting the hostile nature of these fungi if not promptly recognized and treated promptly. Based on the aforementioned case reports, the combination of voriconazole and terbinafine seems promising but more data is needed to define its safety and duration of treatment.

## Conclusions

This case report helps to raise the awareness of medical professionals about this rare opportunistic fungal pathogen that has become increasingly reported in the literature. Our patient had an atypical presentation with rapid clinical deterioration before the fungal pathogen was identified. We believe that vigilant attention to early diagnosis and management could avoid otherwise devastating clinical outcomes. Moreover, the combination of voriconazole and terbinafine is promising with more data needed to define its safety and duration of treatment.
